# Pharmacological effects of *Pugionium cornutum* (L.) Gaertn. extracts on gastrointestinal motility are partially mediated by quercetin

**DOI:** 10.1186/s12906-021-03395-y

**Published:** 2021-09-03

**Authors:** Chencan Su, Haoyu Li, Bang Chen, Cong Li, Chunxiao Zhang, Long Xu, Mei Lan, Yehua Shen

**Affiliations:** 1 Key Laboratory of Synthetic and Natural Functional Molecule of Ministry of Education, College of Chemistry and Materials Science, National Demonstration Center for Experimental Chemistry Education, Northwest University, 229 North Taibai Avenue, Xi’an, 710127 Shaanxi China; 2 College of Chemistry and Chemical Engineering, Yan’an University, Yan’an, 716000 Shaanxi China; 3 Shaanxi Provincial Academy of Environmental Science, Xi’an, 710061 Shaanxi China; 4 Digestive Internal Medicine Department, Shaoxing Paojiang Hospital, Shaoxing, 312000 Zhejiang China

**Keywords:** *Pugionium cornutum* (L.) Gaertn., Gastric smooth muscle cell, Colonic smooth muscle strip, Calcium influx, Quercetin

## Abstract

**Background:**

The majority of global population suffer from various functional gastrointestinal disorders. *Pugionium cornutum* (L.) Gaertn. (PCG) is used to relieve indigestive symptoms in traditional Chinese medicine. However, little is known about the effects of bioactive components from PCG extracts on gastrointestinal motility.

**Methods:**

Crude ethanol extract of PCG (EEP) was prepared from *Pugionium cornutum (L.)* Gaertn. Different solvents were used to prepare fine extracts from EEP, including water extract of PCG (WEP), petroleum ether extract of PCG (PEEP), dichloromethane extract of PCG (DEP) and ethyl acetate extract of PCG (EAEP). Smooth muscle cell model and colonic smooth muscle stripe model were used to test the bioactive effects and mechanisms of different PCG extracts on contraction and relaxation. Diverse chromatographic methods were used to identify bioactive substances from PCG extracts.

**Results:**

EEP was found to promote the relaxation of gastric smooth muscle cell and inhibit the contraction of colonic smooth muscle strip. Among the fractions of EEP, EAEP mainly mediated the relaxation effect by stimulating intracellular calcium influx. Further evidences revealed that EAEP was antagonistic to acetylcholine. In addition, COX and NO-GC-PKC pathways may be also involved in EAEP-mediated relaxation effect. Quercetin was identified as a bioactive compound from PCG extract for the relaxation effect.

**Conclusion:**

Our research supports the notion that PCG extracts promote relaxation and inhibits contraction of gastrointestinal smooth muscle at least partially through the effect from quercetin.

## Background

In modern society, a large proportion of global population suffer from at least one of the functional gastrointestinal disorders due to irregular life style, work pressure or dietary stimulation. In clinics, functional gastrointestinal disorders refer to a group of disorders including gastroparesis, gastroesophageal reflux disease chronic constipation, intestinal pseudo-obstruction, functional dyspepsia, irritable bowel syndrome and chronic constipation [1]. Various acute and chronic symptoms such as dyspepsia, constipation and diarrhea have significant negative impacts on patients’ quality of life by inducing emotional distress [2]. The side effects of targeting drugs are always inevitable, which has become a major concern during treatment. In contrast, functional food for regular uptake is beneficial for the maintenance of healthy status in chronic diseases including gastrointestinal disorders. Therefore, the traditional concept of medicine food homology (MFH) has been widely revisited and developed [3].

The MFH combines the properties of food and drug, including herbs in traditional Chinses medicine [3, 4], fruit and vegetables such as papaya and onion [5]. Notably, herbs have been widely used to treat functional gastrointestinal disorders for a long history with reliable therapeutic effects and tolerable side effects [6]. To evaluate the efficacy and safety more scientifically, herbs have been analysed with modern techniques and applied to clinical trials with appropriate controls [6, 7]. Nowadays, herbs have been used in the therapy of more specific gastrointestinal disease. For example, single herb such as Ren Shen (Radix Ginseng) and Chinese medicine formulae Si-Mo-Tang have been demonstrated as effective therapeutic methods for functional abdominal pain syndrome [8]. In addition, many MFH species are known to regulate metabolism and homeostasis in gastrointestinal system. For instance, astragalus and coix seed have been identified to exert hypolipidemic and hypoglycemic effects and simultaneously enhance immunity [9, 10].

*Pugionium cornutum (L.)* Gaertn. (PCG) is a typical desert plant which is widely distributed in the sand lands of northwest China [11]. As an effective anti-desertification species, the cultivation of PCG has been focused. In addition, its potential food and drug values have been gradually investigated in recent years [12]. As a typical MFH, PCG is considered as a food supplement or a phytomedicine to treat indigestive symptoms [12], and has become a promising candidate in the development of novel gastrointestinal modulator. Notably, the protein content (24.30%) in the dry weight of PCG is much higher than sugar (10.93%) and fat (4.95%) contents, only after dietary fiber content (43.08%) [12] PCG is also known as an ideal dietary source of minerals for its rich potassium and calcium contents [12]. PCG has been demonstrated as a typical MFH species for its potential as a nutritious vegetable and a multi-functional drug in controlling blood lipid level [13] and preventing non-alcoholic fatty liver disease. However, as far as we know, there are very few systematic studies of PCG extract bioactivity on the physiology of digestive system.

Previously, our group and others reported that the water extract of PCG can promote gastrointestinal activity in a non-violent way [12, 14]. However, more bioactive substances could be omitted for their insolubility in water. In this article, the physiological functions of organic solvent extracts from PCG were tested in in vitro models of gastrointestinal smooth muscle. The bioactive substance from PCG extracts was identified to facilitate gastrointestinal motility. Our investigation has provided a new insight into the functional applications of PCG and widely broadened its economic value in drug development.

## Methods

### Chemicals and reagents

Analytic-grade methanol, petroleum ether, dichloromethane, ethyl acetate, dichloromethane and methanol were all purchased from Tianli chemical reagent company, China. Dimethyl sulfoxide(DMSO) (> 99% purity) was purchased from AppliChem. Acetylcholine, methylene blue and indomethacin were obtained from Sigma-Aldrich. Krebs-Henseleit buffer was purchased from Zhongke Technology Company, China. Fetal bovine serum, trypsin and collagenase were purchased from Sigma-Aldrich. Hanks Buffer was purchased from Procell.

### Preparation of PCG extracts

PCG samples were collected from Maowusu desert (also called Mu Us sandy land) in China in August 2015 and stored at − 80 °C. Voucher specimen of PCG was first identified in the field, then confirmed and deposited in the Chinese Medicine Center in School of Life Sciences, Northwest University (by Prof. Dr. Qian Li) under the voucher No. NWU-CCM-00724. The collection of PCG was approved by the Department of Agriculture in Shaanxi Province for the purpose of scientific research with official permission. The collection procedure was carried out in accordance with the Regulations of the People’s Republic of China on Wild Plants Protection.

The roots of fresh PCG were removed and discarded. The remaining aerial parts were washed by distilled water, air dried at 60 °C and milled into fine powder. 5 kg dried powder were mixed with 95% ethanol in a ratio of 1:8 (m/V), heated under reflux for three times (2 h each time) and filtered. The extraction process was repeated once. Filtered supernatant was named as crude ethanol extract of PCG (EEP) and diluted in DMSO at indicated concentrations.

To prepare fine extracts from EEP with different organic solvents, EEP was resuspended in water, petroleum ether, dichloromethane or ethyl acetate, resulting in water extract of PCG (WEP), petroleum ether extract of PCG (PEEP), dichloromethane extract of PCG (DEP) and ethyl acetate extract of PCG(EAEP), respectively. WEP, PEEP, DEP and EAEP were therefore known as fractions of crude EEP, and further tested for their bioactivities separately.

### Animals and ethic approval

Adult Sprague-Dawley (SD) rats weighing 250–350 g were purchased from the lab animal center of Fourth Military Medical University in Xi’an, China. Animals were maintained in standard temperature and humidity conditions with 12 h/12 h light/dark cycle. Animals were fasted with water supply only 24 h before sacrificed for experiments. Both genders were included in experiments. Adult rats were initially anaesthetized with 3% isoflurane for 5 min and then fully anaesthetized with 5% isoflurane in a 3 L chamber to induce euthanasia. The animals were kept in the chamber for 1 min after ceased breathing. Euthanasia was then confirmed by cervical dislocation. Stomach and distal colon were isolated for primary culture. All animal experiments were approved by the Ethics Committee for Animal Experiments in Northwest University (license No. 11375), and performed according to the relevant guidelines. Details can be found in the section of “Ethics approval and consent to participate”.

### Cell isolation and culture

Primary gastric smooth muscle cells (SMCs) were isolated from the stomach tissues of adult SD rats. Tissues were minced and digested at 37 °C by collagen I for 4 h and by trypsin for 1 h. Single cells were obtained by passing through 100 μm cell strainer and cultured in DMEM plus 15% FBS at 37 °C incubator with 5% CO_2_. Medium was changed every 2–3 days.

HEK293 cells were purchased from Beyotime (Catalogue No. C6002). HEK293/MLNR is a transfected HEK293 cell line overexpressing motilin receptor (MLNR), which was previously constructed and stored in our laboratory. HEK293 cells and HEK293/MLNR cells were maintained in the same condition as primary SMCs.

### SMC contraction measurements

SMCs were cultured in 24 well plates. PCG extracts were diluted in DMEM at different concentrations. 0, 20, 40, 60, 80 or 100 mg/ml EEP, EAEP, PEEP, DEP and WEP were used in this test. DMEM was used as negative control. SMCs were treated with these extracts for 1 min at 37 °C, and then washed with PBS, fixed with 40% formaldehyde for 10 min and immersed in 300 μl PBS per well. Fifty cells per group were randomly selected and cell length for each cell was measured under phase contrast microscope (PROVIS, Olympus). Cell contraction rate was calculated by the following formula:

Cell contraction rate (%) = 100% × (Average cell length of control group - Average cell length of experiment group) / Average cell length of control group.

Plus represents contraction and minus represents relaxation.

To investigate the interactive effect of EAEP and acetylcholine (Ach), SMCs were treated with either 100 mg/mL EAEP, 10^− 5^ M Ach, 10^− 3^ M Ach or 100 mg/mL EAEP plus 10^− 3^ M Ach. DMEM only were used as negative control. The treatment and data analysis were performed as described above.

### Measurements of intracellular free calcium concentration

SMCs, HEK293 cells and HEK293/MLNR cells were seeded at 10000 cells per 25 μL in one well of 384 well plate and cultured overnight. The concentration of free calcium was measured by MD Ca^2+^ 5 Assay Kit (Molecular Devices). Briefly, culture medium was aspirated from the wells. 25 μL fresh medium and 25 μL loading buffer from the kit were added into the well for 1 h incubation at 37 °C. After incubation, 12.5 μL PCG extract at indicated concentration was added, and intracellular free calcium concentration was recorded by Flexstation 3 plate reader (Molecular Devices). For SMCs, cells were treated with 0, 20, 40, 60, 80 or 100 mg/mL EAEP. For HEK293 and HEK293/MLNR cells, cells were treated with 0, 20, 60 or 100 mg/mL EAEP.

### Preparation of colonic smooth muscle strips

The distal colon from adult SD rats were isolated and excised into 1.5 cm strips. Mesenteric and serous membranes were removed to expose smooth muscle. The segments were then washed in Krebs-Henseleit buffer (M&C Gene Technology Ltd., China) and suspended in an organ bath filled with 10 mL Krebs-Henseleit buffer and bubbled with 95% O_2_ and 5% CO_2_ at 37 °C. The cleaned strips were hooked to an isometric force transducer with an initial tension of 1 g and then equilibrated for 1 h with replaced Krebs-Henseleit buffer every 20 min. Contraction force was amplified and recorded by LabChart 8 biological function experimental system (Spirometry, AD instruments).

### Measurements of colonic smooth muscle strip contraction

After equilibration, 10, 20, 40, 60, 80 and 100 mg/ml EEP, EAEP, PEEP and DEP were prepared, and 50 μL extract from the lowest dose was added subsequently into 10 mL organ bath to treat the prepared strips. The effect was recorded for a few minutes before washout. The strips were washed once in Krebs-Henseleit buffer before applied to the next dose.

To further investigate the plausible mechanism by which PCG extracts regulate colonic smooth muscle contraction, prepared strips were pre-treated with acetylcholine, methylene blue or indomethacin and equilibrated. After then, 50 μL 100 mg/mL EEP was added into 10 mL organ bath and contraction tension was recorded.

### Identification of bioactive compounds in EAEP

EAEP was dissolved in indicated organic solvent and loaded onto a silica gel column. The column was eluted in tandem with dichloromethane: methanol (V:V = 80:1), dichloromethane: methanol (V:V = 60:1), dichloromethane: methanol (V:V = 40:1), dichloromethane: methanol (V:V = 20:1) and methanol. Elutes from half column were counted as one batch and resulted in two batches. Sub-fractions were combined by thin-layer chromatography in each batch, and 10 fractions per batch were obtained. The fractions were used for the measurement of colonic smooth muscle contraction as described above. Active fractions were further separated by HPLC (D-2000 Elite, Hitachi) for bioactivity analysis. The final bioactive compound was identified by UV spectroscopy (UV-2550, Shimadzu), FT-IR spectroscopy (TENSOR27, Bruker), NMR (WNMR-1, Varian) and mass spectrometry (QP-2010 Plus, Shimadzu).

### Statistics

All results were demonstrated as mean ± standard error of mean (SEM). At least three independent replicates were included, and sample numbers were indicated in each table or figure legend. Two-tailed t-test was performed for statistical analysis and *p*-value less than 0.05 (*p* < 0.05) was considered as significant.

## Results

### EEP promotes the relaxation of SMC

**Fig. 1:**
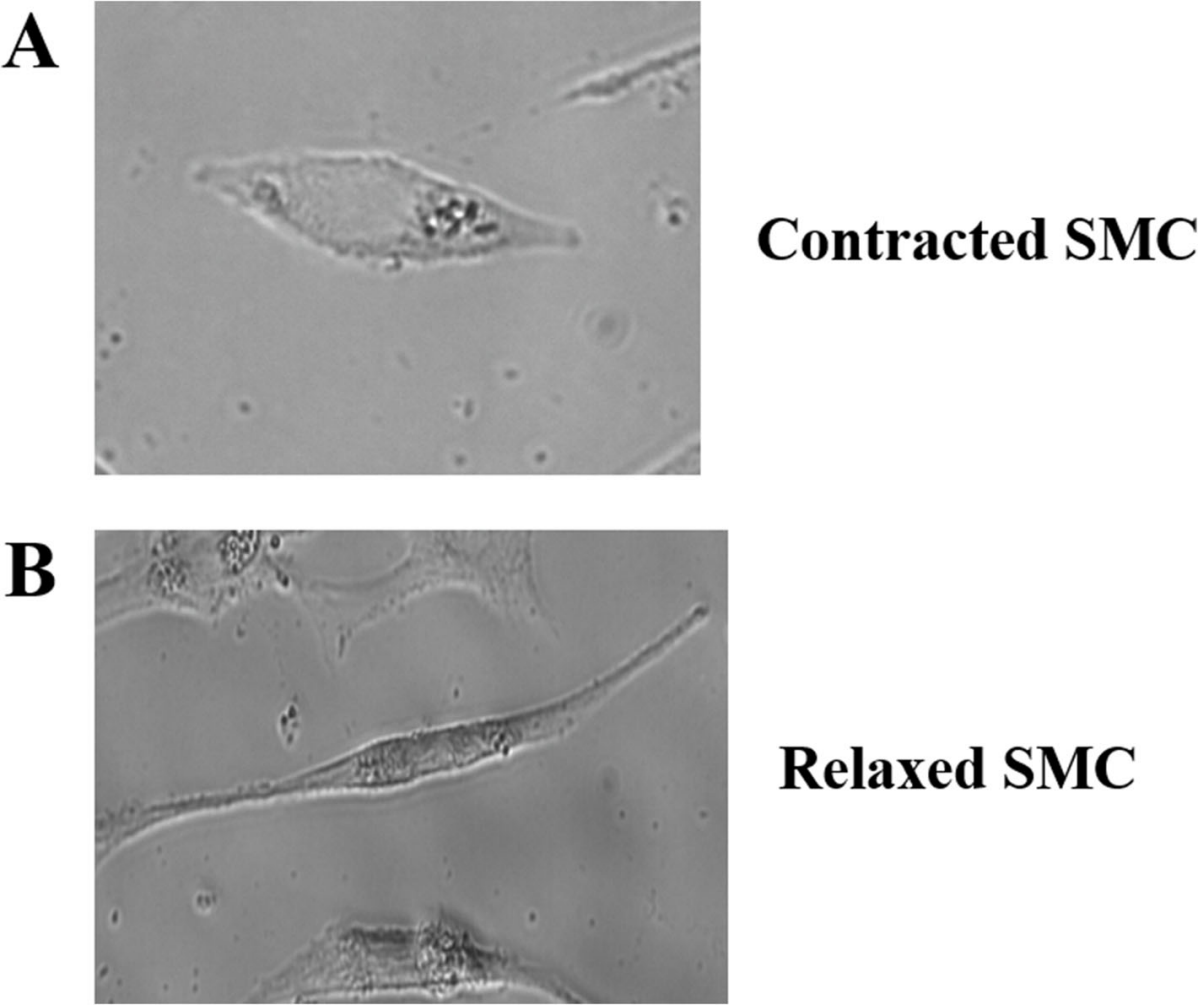
The morphology of cultured SMCs in the forms of contraction (**A**) and relaxation (**B**) under phase-contrast microscope

Two morphological forms were observed in SMCs isolated from the stomach of SD rats: contracted form (Fig. 1a) or relaxed form (Fig. 1b). After the treatment of EEP, the average length of SMCs was significantly increased in a dose-dependent manner (Table 1), reflecting an obvious relaxation effect. Among the fine extracts of EEP, a even stronger effect of relaxation was observed in EAEP, and a lower extent in WEP (Table 2 and Table 3). Instead, the treatments with PEEP and DEP showed no significant difference on SMC contraction (Table 4 and Table 5). To unravel whether EAEP played an interactive role with acetylcholine, SMCs were treated either with EAEP alone, or with two different doses of acetylcholine (10^− 5^ and 10^− 3^ M), or with the combination of EAEP and acetylcholine (Table 6). As is shown in Table 6, acetylcholine strongly promoted contraction, while this effect can be suppressed by EAEP (Table 6). Our data suggested that EAEP is the major functional fraction of EEP to relax SMC in an acetylcholine-dependent pattern.

**Table 1:** The effects of EEP on contractile response of gastric SMCs

EEP concentration (mg/ml)	SMC average length (μm)	Contraction rate (%)
0	80.56 ± 4.81	0
20	82.73 ± 9.24	−2.26
40	92.35 ± 15.17*	−14.64
60	92.88 ± 7.73*	−15.29
80	94.80 ± 14.25*	−17.68
100	97.01 ± 5.53*	−20.43

*N* = 50, * *P* < 0.05, data (mean ± SEM) were analyzed by ANOVA

**Table 2:** The effects of EAEP on contractile response of gastric SMCs

EAEP concentration (mg/ml)	SMC average length (μm)	Contraction rate (%)
0	84.62 ± 4.76	0
20	89.06 ± 13.07	− 5.24
40	95.95 ± 6.00*	−13.39
60	101.97 ± 4.18*	−20.50
80	112.57 ± 18.17*	−33.03
100	111.28 ± 12.43*	−31.51

*N* = 50, * *P* < 0.05, data (mean ± SEM) were analyzed by ANOVA

**Table 3:** The effects of WEP on contractile response of gastric SMCs

WEP concentration (mg/ml)	SMC average length (μm)	Contraction rate (%)
0	83.47 ± 8.49	0
20	85.24 ± 11.21	−2.12
40	86.53 ± 9.96	−3.66
60	88.28 ± 10.89	−5.76
80	89.62 ± 14.07	−7.36
100	97.14 ± 9.02*	−16.38

*N* = 50, * *P* < 0.05, data (mean ± SEM) were analyzed by ANOVA

**Table 4:** The effects of PEEP on contractile response of gastric SMCs

PEEP concentration (mg/ml)	SMC average length (μm)	Contraction rate (%)
0	84.93 ± 8.78	0
20	86.41 ± 10.45	−1.75
40	87.31 ± 8.35	−2.81
60	82.87 ± 6.14	2.42
80	85.63 ± 12.01	−0.82
100	90.30 ± 15.28	−6.33

*N* = 50, not significant, data (mean ± SEM) were analyzed by ANOVA

**Table 5:** The effects of DEP on contractile response of gastric SMCs

DEP concentration (mg/ml)	SMC average length (μm)	Contraction rate (%)
0	85.18 ± 5.75	0
20	83.59 ± 9.89	1.87
40	90.17 ± 10.86	−5.86
60	82.45 ± 12.99	3.21
80	81.19 ± 11.82	4.68
100	91.60 ± 8.79	−7.54

*N* = 50, not significant, data (mean ± SEM) were analyzed by ANOVA

**Table 6:** The interactive effects of EAEP and acetylcholine (Ach) on contractile response of SMCs

Treatment	SMC average length (μm)	Contraction rate (%)
Control	87.51 ± 6.06	0
EAEP	101.04 ± 6.91*	−15.47
Ach-5	76.01 ± 4.89*	13.14
Ach-3	70.66 ± 6.65*	19.25
EAEP+Ach-5	93.42 ± 13.73	−6.75
EAEP+Ach-3	89.05 ± 11.38	−1.76

*N* = 50, * *P* < 0.05, data (mean ± SEM) were analyzed by ANOVA. EAEP: 60 mg/ml; Ach-5: 10^−5^ M; Ach-3: 10^−3^ M

### EAEP increases the intracellular calcium concentration

**Fig. 2:**
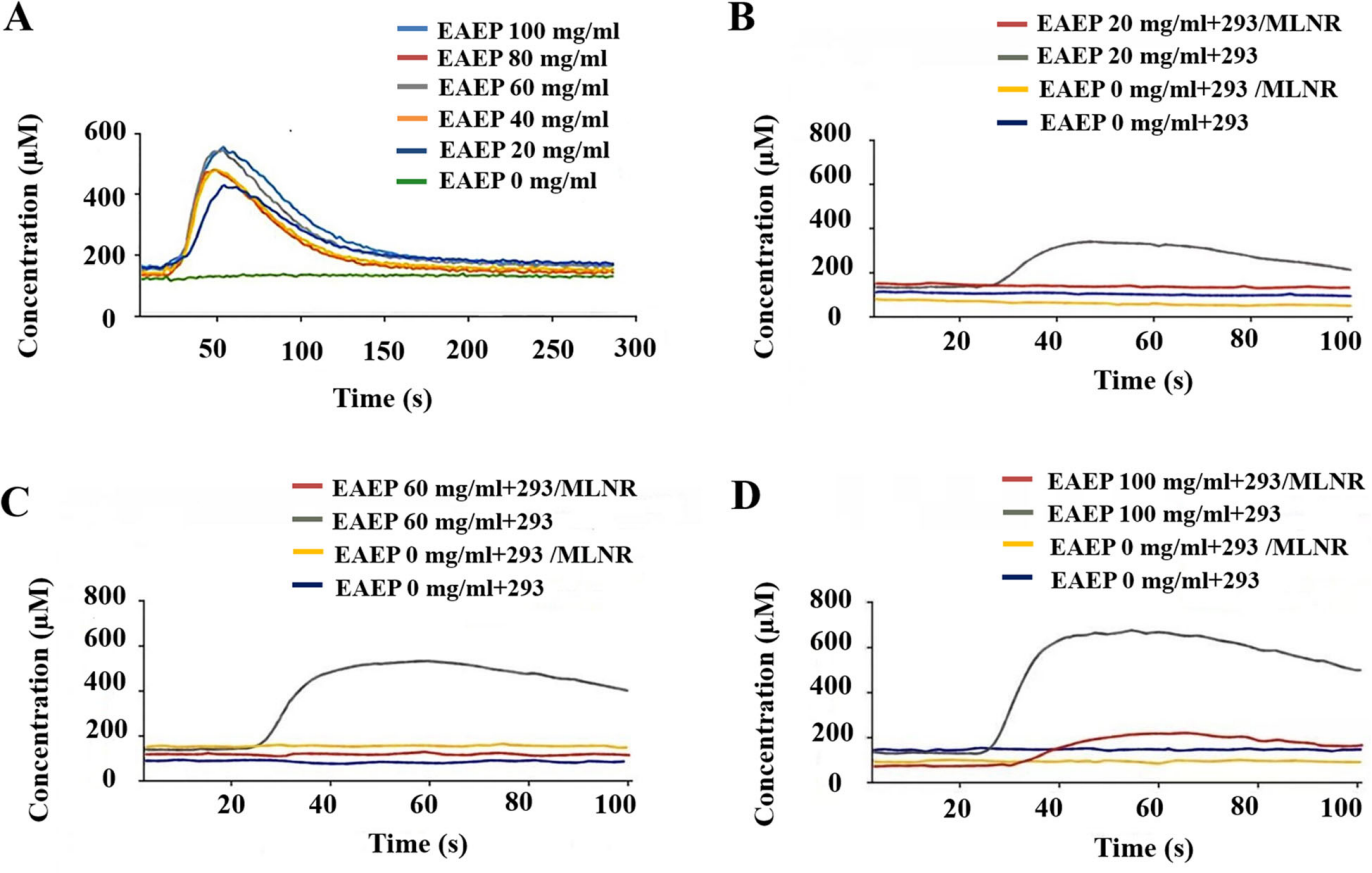
Stimulation of intracellular free calcium influx upon EAEP treatment in SMCs (**A**) or HEK293/HEK293 MLNR cells (**B**-**D**). 0, 20, 40, 60, 80 or 100 mg/ml EAEP were used to treat SMCs (**A**). 20 mg/ml EAEP (**B**), 60 mg/ml EAEP (**C**) or 100 mg/ml EAEP (**D**) were used to treat HEK 293 and HEK293 MLNR cells. X-axis: time after treatment (s); Y-axis: calcium concentration in μM

Calcium ion is one of the most important modulators of various enzymes, channel proteins and signaling proteins. As a second messenger, calcium plays multiple physiological roles. Intracellular free calcium concentration can reflect the cellular response to external drug and environmental stimulations. In SMCs, the upregulation of intracellular free calcium concentration leads to cell contraction [15–17]. It is speculated whether PCG extract has an impact on intracellular free calcium accumulation. To test this hypothesis, EAEP was selected for its best performance in promoting contraction (Tables 2, 3, 4 and 5). As measured in Fig. 2a, intracellular free calcium concentration in SMCs was sharply enhanced to the peak upon EAEP treatment within 1 min (Fig. 2a). The effect was shown in a dose-dependent manner within the range of 20–100 mg/mL (Fig. 2a). After 3 min, the calcium concentration was gradually decreased to baseline level (Fig. 2a). The decrease is probably due to the negative feedback loop to avoid constant stimulation in the cell [16]. In the next step, we attempted to understand the mechanisms behind EAEP-mediated effect on intracellular free calcium stimulation. Motilin is one of the most important factors in controlling the inter-digestive migrating contractions [18]. To test if the effect of EAEP was mediated by motilin, HEK293/MLNR knock-in cell line with overexpressed motilin receptor was used for experiments, and wildtype HEK293 cell was used as control. In HEK293 cells, the effect of EAEP was similar to that of SMCs in a dose-dependent pattern (Fig. 2b-d). Surprisingly, almost no effect on intracellular free calcium concentration was observed in HEK293/MLNR cells compared to wildtype HEK293 cells upon EAEP treatment (Fig. 2b-d). This result indicates that the effect of EAEP on intracellular free calcium accumulation is independent of motilin pathway, but is driven by other mechanisms.

### EAEP inhibits the contraction of colonic smooth muscle strip

**Fig. 3:**
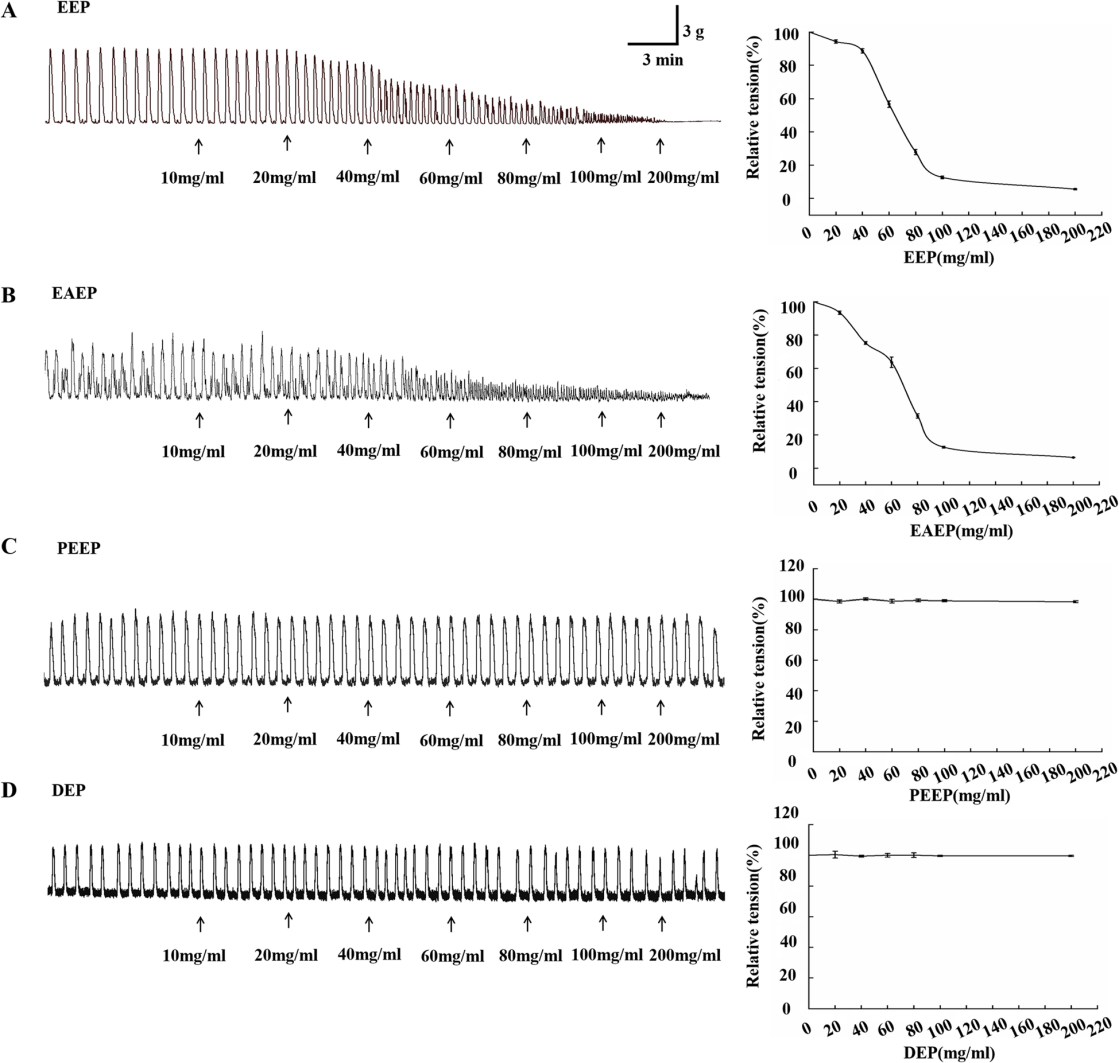
EAEP fraction of EEP is the major fraction to inhibit the contraction of colonic smooth muscle in a dose-dependent manner. 10, 20, 40, 60, 80, 100 and 200 mg/ml of each fraction was subsequentially added to colonic smooth muscle stripe. Left panel: original records of colonic smooth muscle contraction upon treatment with indicated doses of EEP (**A**), EAEP (**B**), PEEP (**C**) and DEP (**D**). X-axis: time after treatment (min); Y-axis: contractile tension force (g). Right panel: correlation curve from the data in left panel

Colonic smooth muscle stripe is a commonly used tissue model for contractility study. A wide concentration range (0, 10, 20, 40, 60, 80, 100 and 200 mg/ml) of different extracts were prepared and tested for their effects on the contraction of colonic smooth muscle strips. The results demonstrated that EEP inhibited strip contractility in a dose-dependent pattern (Fig. 3a). Among the fine extracts of EEP, EAEP demonstrated a similar inhibitory effect of EEP (Fig. 3b). Instead, PEEP and DEP did not change the contraction pattern of the strip (Fig. 3c and d, respectively). The results indicated that EAEP is the main fraction of EEP with contraction-inhibitory activity in colonic smooth muscle tissue. This in vitro tissue model confirms our findings in cultured SMCs, that EAEP is the key EEP fraction to promote relaxation and inhibit contraction, while PEEP and DEP fractions are non-effective.

### The inhibitory effect of EEP is mediated by several mechanisms

**Fig. 4:**
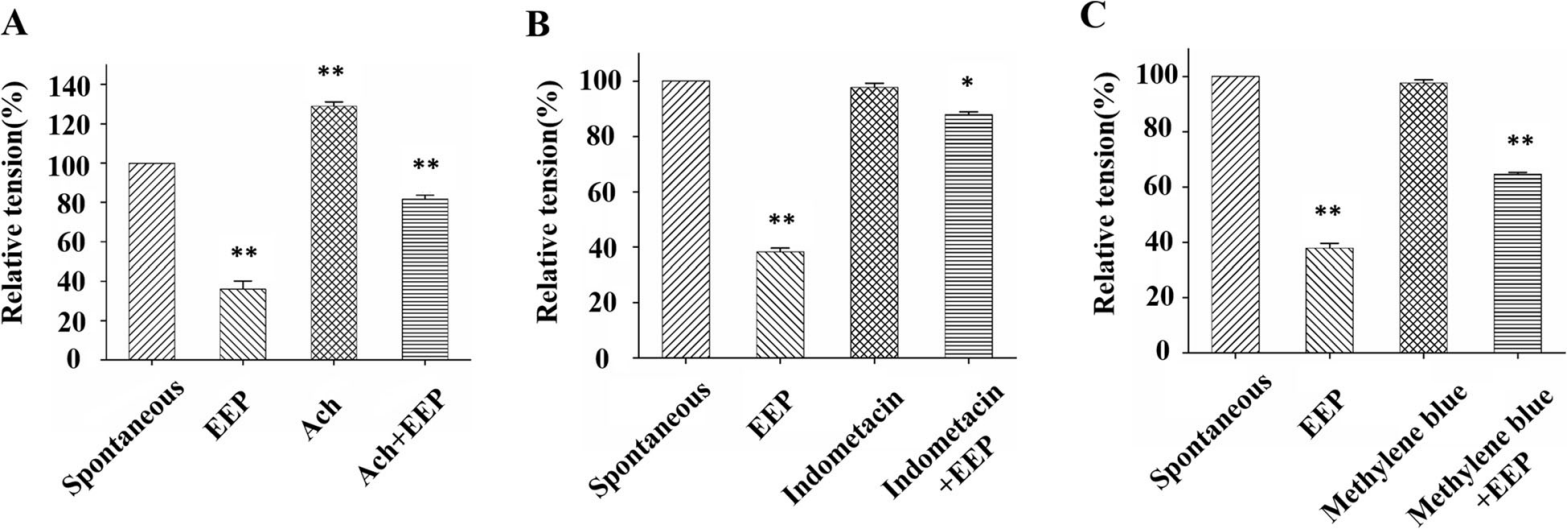
Interactive effects of EEP with acetylcholine, methylene blue or indomethacin on the contractile response of colonic smooth muscle. Relative tension of colonic smooth muscle contraction was measured after pre-treatment with acetylcholine (**A**), indomethacin (**B**) or methylene blue (**C**), and subsequent treatment with EEP. Spontaneous:spontaneous contraction; Ach: acetylcholine. * *P* < 0.05, **P < 0.01, data (mean ± SEM) were analyzed by ANOVA

To understand the underlying mechanisms how EEP promotes relaxation and suppresses contraction, EEP was tested in several classic modulating pathways of gastrointestinal motility. First, acetylcholine is a neurotransmitter and activates smooth muscle contraction [19]. The addition of EEP was found to impair acetylcholine treatment-induced contraction with an inhibition rate of 35.57 ± 3.35% in colonic smooth muscle stripe model (Fig. 4a). In comparison, in control group without acetylcholine treatment, the inhibition rate was 61.17 ± 3.32%. The antagonistic effect of EEP to acetylcholine is consistent with the fact that the major effective fraction EAEP from EEP stimulates intracellular free calcium concentration (Fig. 2a-d), as acetylcholine is known to block calcium channel and reduce calcium influx [20].

Second, prostaglandins are a group of regulating lipid compounds with multiple functions including the stimulation of smooth muscle contraction [21]. Indomethacin is a non-selective inhibitor of cyclooxygenase (COX) and further inhibits the synthesis of postagladin [22]. In our experiments, no change of contractility was observed in indomethacin pre-treated strips (Fig. 4b). Subsequent treatment with EEP suppressed the contraction with the inhibition rate of 11.24 ± 8.23%, which was significantly lower than the control group (61.17 ± 3.32%). The data indicated an antagonistic effect of EEP to indomethacin.

Third, nitric oxide (NO) is the first messenger molecule to promotes smooth muscle relaxation through NO-guanylate cyclase (GC)-protein kinase C (PKC) pathway [23]. In our experiments, no effect on contractility was found in strips pre-treated with methylene blue, a typical GC inhibitor (Fig. 4c). The subsequent addition of EEP reduced the contraction with the inhibition rate to 33.64 ± 5.54%, comparing with 61.17 ± 3.32% in control group, showing a similar but milder antagonistic effect to indomethacin.

To conclude, EEP may inhibit smooth muscle contraction through acetylcholine receptor, COX and NO-GC-PKC pathways.

### Identification of quercetin as a bioactive substance in PCG extract

**Fig. 5:**
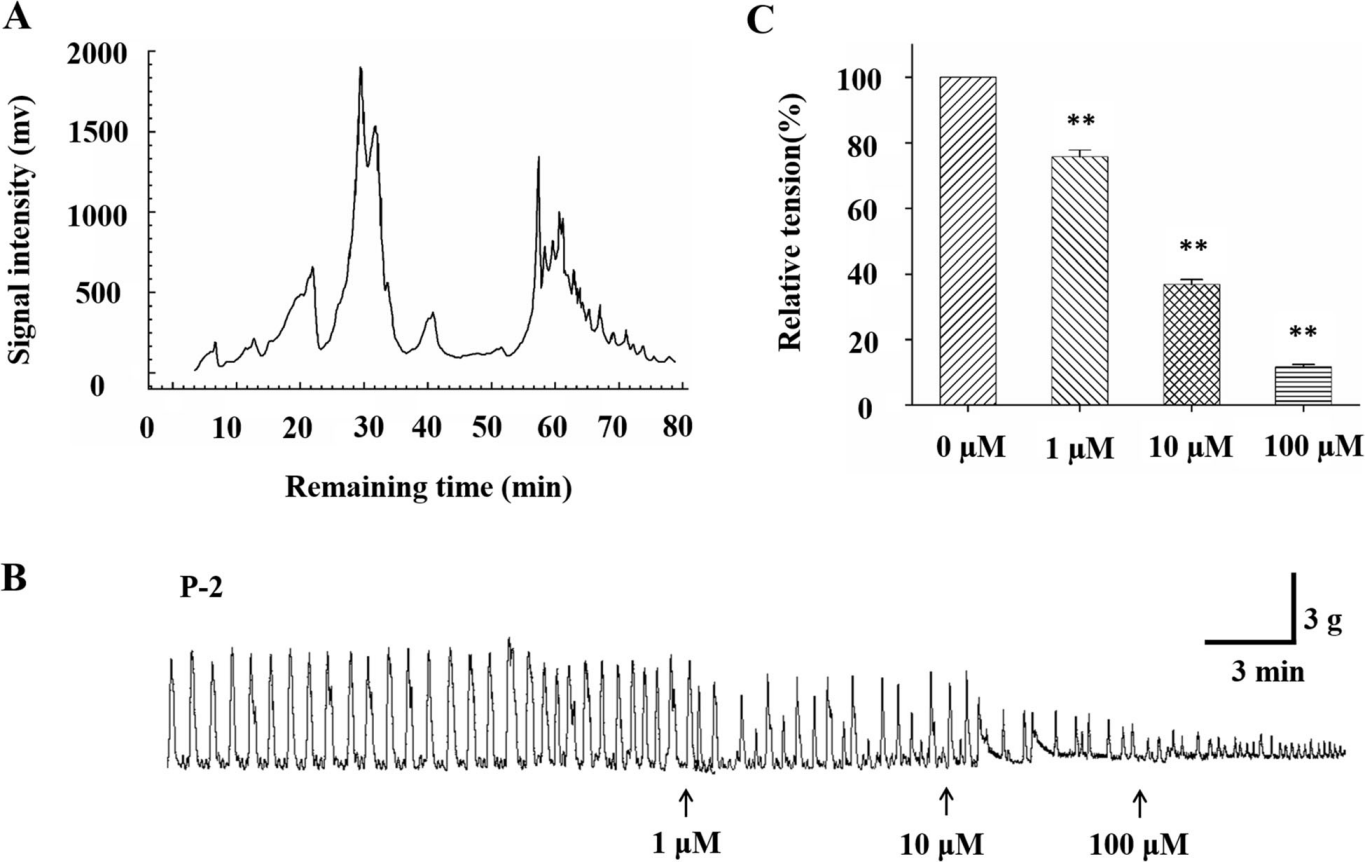
Identification of bioactive compound in PCG extracts. After bioactive test, the active fractions were separated to single compounds by HPLC (**A**). The compound collected between 25 and 35 min were confirmed as a bioactive substance by relaxing colonic smooth muscle (**B** and **C**) and named as P-2

**Fig. 6:**
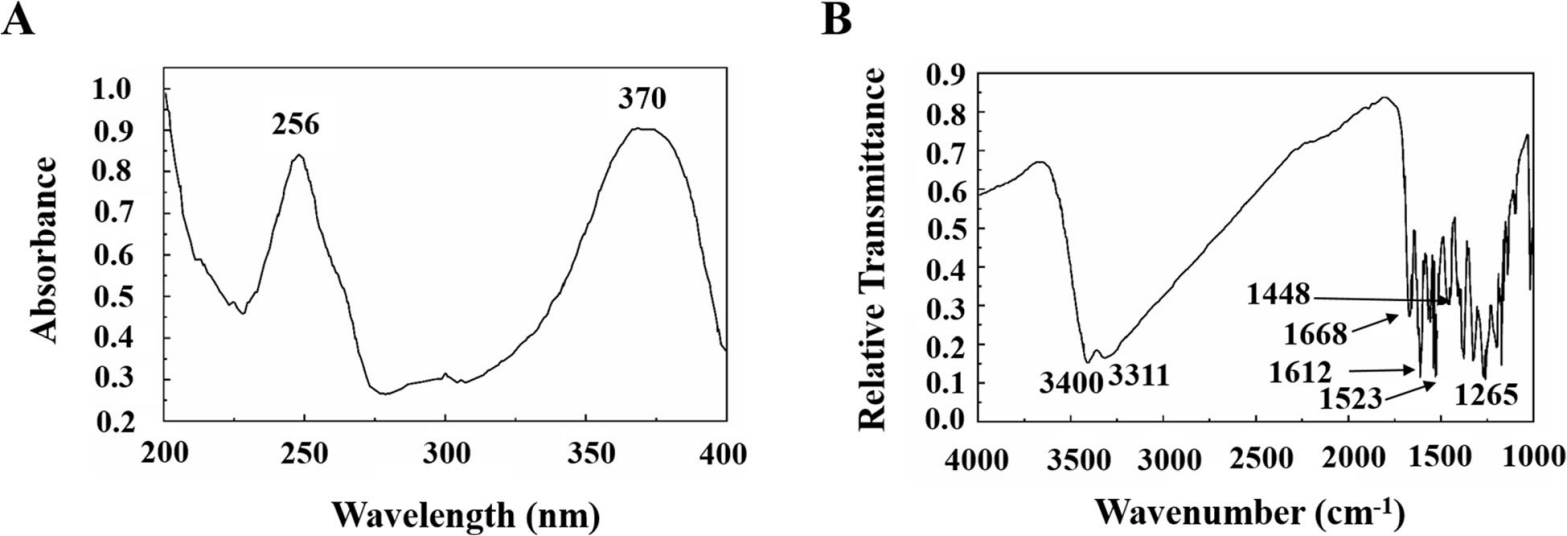
Additional spectral analysis of compound P-2-1 purified from EAEP. (**A**) UV-spectrum; (**B**) FT-IR spectrum

It is difficult to control the quality of bioactive components in plant extracts from batch to batch. Therefore, identification and characterization of bioactive compound is critical in food and drug development. Column chromatography, high performance liquid chromatography (HPLC) and thin-layer chromatography (TLC) are common chromatographic techniques to isolate bioactive compounds from plant crude extracts [24, 25]. We attempted to isolate bioactive substance from PCG extracts using chromatographic methods. First, column chromatography and TLC were used to isolate crude fractions from EAEP which were proved to be active in relaxing SMC and colonic smooth muscle strip. Eight of 20 fractions were found to be able to induce relaxation (Table 7). The active fractions were combined and further isolated by HPLC with the following conditions: distilled water was used as mobile phase Solvent A and methanol as mobile phase Solvent B. Both Solvent A and B were degassed for 30 min with ultrasonication. Flow speed was kept at 1.0 mL/min. For gradient elution, 10–80% Solvent B was used in 0–60 min, and 80–100% Solvent B was used in 60–80 min. The results showed three main peaks within 80 min, among which peak 2 is the highest (Fig. 5a). Sub-fractions between 1 and 25 min (P-1), 25–35 min (P-2) and 35–80 min (P-3) were collected respectively. Only P-2 sub-fraction demonstrated bio-activity in strip relaxation (Fig. 5b). P-1 and P-3 sub-fractions were inactive (data not shown). Furthermore, P2- was concentrated and crystallized to obtain the pure compound P-2-1. After UV-spectrum (Fig. 6a), FT-IR spectrum (Fig. 6b), mass spectrometry, ^1^H-NMR spectrum and ^13^C-NMR spectrum analysis, the following data were collected.

**Table 7:** The effects of different sub-fractions from EAEP on colonic smooth muscle contractility. ‘+’ means positive effect and ‘-’ means negative effect (*N* = 10)

No. in batch 1	Relaxation effect	No. in batch 2	Relaxation effect
1	–	1	–
2	+	2	–
3	+	3	+
4	+	4	+
5	–	5	+
6	+	6	+
7	–	7	–
8	–	8	–
9	–	9	–
10	–	10	–

Compound P-2-1: yellow powder; mp 314 °C–316 °C; dissolved in ethanol, methanol and diluted alkali solution; UV(λmax) nm: 256, 370sh (MeOH); IR(KBr: Vmaxcm^− 1^): 3400, 3311, 1668, 1612, 1523, 1448, 1265; 1H-NMR(CD_3_OD) δppm: 6.22, 6.44, 6.90, 6.92, 7.71, 9.37, 9.43, 9.65, 10.84, 12.53; ^13^C-NMR(CD_3_OD) δppm: 93.78, 98.60, 103.43, 115.46, 116.03, 120.39, 122.37, 136.18, 145.48, 147.20, 148.13, 156.55, 161.15, 164.31, 176.27; with sodium TOF-MSm/z: 303.0492.

The compound P-2-1 was identified as quercetin, of which the characters were previously reported [26].

## Discussion

As an important anti-desertification plants, PCG shows far more potentials beyond environmental protection. As a typical MFH herb, it is not only historically used in traditional Chinese medicine formulae for the treatment of gastrointestinal disorders, but also favored for its minimal side effects. This paper demonstrated how PCG extract promoted relaxation and inhibited contraction of smooth muscle in in vitro cell and tissue model of gastrointestinal system.

The extraction method is one of the key aspects for the isolation of bioactive components from complex plant extracts. Although petroleum ether, dichloromethane, ethyl acetate and water are commonly-used solvents for plant extraction, their extracts may contain different bioactive components and exert even opposite functions due to their distinct polarities, as reported by others [27]. In the case of PCG, ethyl acetate is the best solvent to retrieve the active substances for relaxing gastrointestinal smooth muscle.

In recent decades, thousands of phytomedical plants have been validated in regulating a much broader range of bioactive hormones and receptors in digestive system such as motilin, somatostatin and P substance [28–30]. Since our data do not support the involvement of motilin in EAEP-mediated effects (Fig. 2b-d), other calcium-dependent mechanisms such as cytoskeletal α-smooth muscle actin (α-SMA), α5β1 integrin, and integrin-mediated cell-extracellular matrix may be worthwhile to test in order to figure out which pathways are influenced by EAEP. In addition, as in vivo model of intracellular calcium measurement on eccentric contractions has been well established, it is meaningful to examine whether EAEP function on intracellular calcium regulation can be confirmed in animal model.

Our data showed that PCG extract modulated gastrointestinal motility through interactions with acetylcholine, COX and NO-GC-PKC pathways (Fig. 4a-c). In the future, it can be interesting to identify more specific target in these pathways. For example, indomethacin was used as a non-selective COX inhibitor in our experiments. COX-1 is constitutively expressed in gastrointestinal tract for physiological functions, while COX-2 is associated with pathological inflammation. Specific inhibitors of COX-1 and COX-2 can distinguish the two biological processes [31]. It can be of great value to discover novel compounds from PCG extracts which target specifically on COX-1 or COX-2 activity.

The bioactive natural products have been isolated and characterized from plant extracts for a long history. Many of them were used as potent drug candidates with higher efficacy and reduced adverse effects. Our results showed that quercetin, one of the effective substances in PCG extracts, mediated PCG’s function in smooth muscle relaxation (Figs. 5 and 6). This will definitely enhance the accuracy, effectiveness and safety of PCG–based drug development. In the future, it will be appealing to isolate other bioactive substances other than quercetin from PCG extracts which may also regulate gastrointestinal motility in vitro and in vivo.

Based on the food safety properties, bioactive compounds and extracts from PCG can also be developed directly as dietary supplement to improve gastrointestinal function. In addition, it is also possible to explore bioactivities of PCG extracts in the therapy of a broader context of diseases such as cancer in digestive system.

## Conclusion

**Fig. 7:**
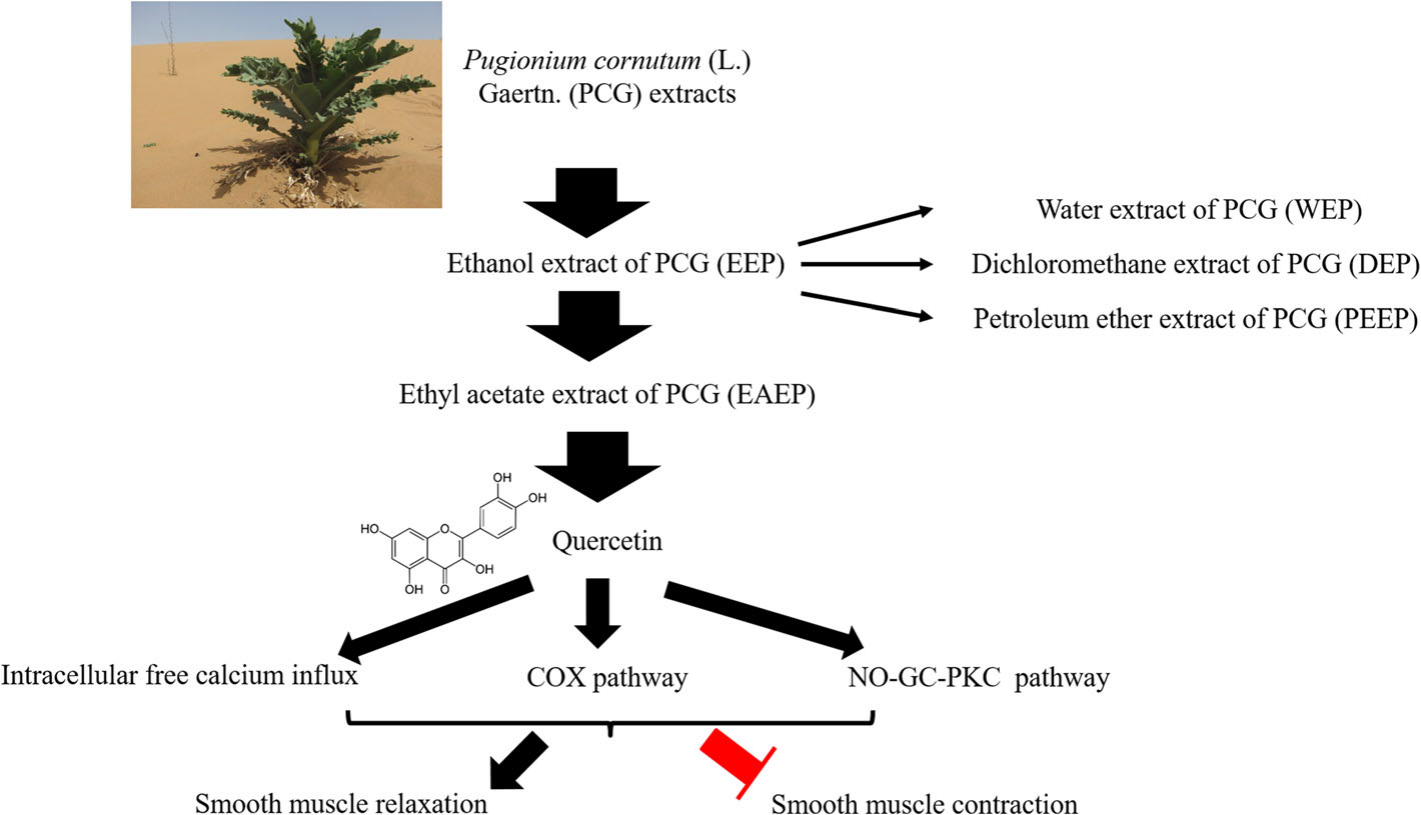
Graphic summary. In the crude EEP extract, the EAEP fraction, but not WEP, DEP or PEEP fractions, can mediate the effects on gastrointestinal motility. One of the key bioactive substances in EAEP is identified as quercetin, which may mediate the functions of EAEP in promoting relaxation and inhibiting contraction of gastrointestinal smooth muscle. The underlying molecular mechanisms involve the regulation of intracellular calcium influx, COX pathway and NO-GC-PKC pathway

We focused on the functions of PCG extracts on gastrointestinal motility in cell model and tissue model in vitro. EEP was found to promote the relaxation of gastric SMC and inhibit the contraction of colonic smooth muscle strip. Among fractions of EEP, EAEP, but not other fractions, was identified to mediate the relaxation effect. The underlying mechanisms of EAEP involved the stimulation of intracellular calcium influx, the antagonistic effect to acetylcholine, and the regulation of COX and NO-GC-PKC pathways. In addition, quercetin was identified as a bioactive compound in PCG extracts. The key information is summarized in Fig. 7. Our results indicate that PCG extracts can be potentially used for drug development in treating gastrointestinal disorders.

## Data Availability

The datasets used or analyzed during the current study are available from the corresponding author on reasonable request.

## Abbreviations

COX: Cyclooxygenase
DEP: Dichloromethane extract of *Pugionium cornutum* (L.) Gaertn
EAEP: Ethyl acetate extract of *Pugionium cornutum* (L.) Gaertn
EEP: Crude ethanol extract of *Pugionium cornutum* (L.) Gaertn
GC: Nitric oxide-guanylate cyclase
HPLC: High performance liquid chromatography
NO: Nitric oxide
PCG: *Pugionium cornutum* (L.) Gaertn
PEEP: Petroleum ether extract of *Pugionium cornutum* (L.) Gaertn
PKC: Protein kinase C
SEM: Standard error of mean
SMC: Gastric smooth muscle cell
TLC: Thin-layer chromatography
WEP: Water extract of *Pugionium cornutum* (L.) Gaertn
